# Differences in the Serum Nonesterified Fatty Acid Profile of Young Women Associated with a Recent History of Gestational Diabetes and Overweight/Obesity

**DOI:** 10.1371/journal.pone.0128001

**Published:** 2015-05-26

**Authors:** Marina Fugmann, Olaf Uhl, Christian Hellmuth, Holger Hetterich, Nora N. Kammer, Uta Ferrari, Klaus G. Parhofer, Berthold Koletzko, Jochen Seissler, Andreas Lechner

**Affiliations:** 1 Diabetes Research Group, Medizinische Klinik und Poliklinik IV, Klinikum der Universität München, Ludwig-Maximilians-Universität München, Munich, Germany; 2 Clinical Cooperation Group Type 2 Diabetes, Helmholtz Zentrum München, Munich, Germany; 3 German Center for Diabetes Research (DZD), Munich, Germany; 4 Division of Metabolic and Nutritional Medicine, Dr. von Hauner Children’s Hospital, Klinikum der Universität München, Ludwig-Maximilians-Universität München, Munich, Germany; 5 Institute for Clinical Radiology, Klinikum der Universität München, Ludwig-Maximilians-Universität München, Munich, Germany; 6 Medizinische Klinik und Poliklinik II, Klinikum der Universität München, Ludwig-Maximilians-Universität München, Munich, Germany; GDC, GERMANY

## Abstract

**Background:**

Nonesterified fatty acids (NEFA) play pathophysiological roles in metabolic syndrome and type 2 diabetes (T2D). In this study, we analyzed the fasting NEFA profiles of normoglycemic individuals at risk for T2D (women with a recent history of gestational diabetes (GDM)) in comparison to controls (women after a normoglycemic pregnancy). We also examined the associations of NEFA species with overweight/obesity, body fat distribution and insulin sensitivity.

**Subjects and Methods:**

Using LC-MS/MS, we analyzed 41 NEFA species in the fasting sera of 111 women (62 post-GDM, 49 controls). Clinical characterization included a five-point oral glucose tolerance test (OGTT), biomarkers and anthropometrics, magnetic resonance imaging (n = 62) and a food frequency questionnaire. Nonparametric tests with Bonferroni correction, binary logistic regression analyses and rank correlations were used for statistical analysis.

**Results:**

Women after GDM had a lower molar percentage of total saturated fatty acids (SFA; 38.55% vs. 40.32%, p = 0.0002) than controls. At an explorative level of significance several NEFA species were associated with post-GDM status (with and without adjustment for body mass index (BMI) and HbA1c): The molar percentages of 14:0, 16:0, 18:0 and 18:4 were reduced, whereas those of 18:1, 18:2, 20:2, 24:4, monounsaturated fatty acids (MUFA), polyunsaturated fatty acids (PUFA) and total n-6 NEFA were increased. BMI and the amount of body fat correlated inversely with several SFA and MUFA and positively with various PUFA species over the whole study cohort (abs(ρ)≥0.3 for all). 14:0 was inversely and BMI-independently associated with abdominal visceral adiposity. We saw no correlations of NEFA species with insulin sensitivity and the total NEFA concentration was similar in the post-GDM and the control group.

**Conclusion:**

In conclusion, we found alterations in the fasting NEFA profile associated with a recent history of gestational diabetes, a risk marker for T2D. NEFA composition also varied with overweight/obesity and with body fat distribution, but not with insulin sensitivity.

## Introduction

Several lines of evidence suggest that NEFA are involved in the pathogenesis of T2D via a reduction of insulin sensitivity and the promotion of pancreatic beta cell apoptosis and dysfunction [1–3]. Particularly in the context of metabolic syndrome, NEFA might represent an important link between obesity and insulin resistance [4, 5]. However, it is not entirely clear whether elevated serum NEFA levels in individuals with T2D or metabolic syndrome are a primary disease-inducing alteration or a secondary change [6, 7].

Different NEFA species have distinct effects on insulin sensitivity, beta cell function and tissue inflammation in experimental setups [8]. SFA, for instance, are able to interact with toll-like receptor 4 (TLR-4) to induce proinflammatory signaling [9], whereas PUFA, such as docosahexaenoic acid (22–6 n-3), inhibit these pathways, e.g., by binding to G protein-coupled receptor 120 [10].

Previous studies have shown differences in the serum NEFA composition between individuals with T2D or metabolic syndrome and controls [11, 12]. We wanted to test the hypothesis that the serum NEFA profile is also altered in a specific cohort of young, normoglycemic individuals with a high risk for T2D, namely in women after GDM. We chose a recent history of GDM to select the at-risk cohort because no reliable biomarkers are available to identify T2D at-risk subjects while they are still normoglycemic. Women with GDM during a recent pregnancy however have an about 10-fold increased risk for subsequent T2D within 10 years and therefore represent a suitable population to address our research question [13, 14]. Women after a normoglycemic pregnancy were included in the study as controls. We also examined the associations of the fasting NEFA profile with body fat distribution and insulin resistance.

## Subjects and Methods

### Study population

Between November 2011 and December 2013 147 women were consecutively recruited within the prospective Prediction, Prevention and Subclassification of Type 2 Diabetes (PPS-Diab) cohort study [15]. Gestational diabetes and normoglycemia during pregnancy were diagnosed with a 75g OGTT between the 24^th^ and the 28^th^ week of gestation following the IADPSG criteria [16]. For this analysis, 111 women (62 cases after GDM and 49 controls) were included due to normoglycemia at the baseline visit between 3 and 16 months after delivery. Over 90% of the study participants were Caucasian. Exclusion criteria for this study were chronic diseases requiring medication except for hypothyroidism. Hormonal contraception was also permitted. Written informed consent was obtained from each subject and the study was approved by the ethics committee of the Ludwig-Maximilians-Universität München.

### Anthropometric and clinical measurements

Weight and fat mass were measured using a bioelectrical impedance analysis (BIA) scale (Tanita BC-418, Tanita Corporation, Tokyo, Japan). BMI was calculated as the weight (in kilograms) divided by height squared (in meters). Hip and waist circumferences (WC) were assessed by tape measurements. Daily physical activity (m/d) was measured by a pedometer (AiperMotion 440, Aipermon GmbH & Co. KG, Munich, Germany). A 5-point 75 g OGTT was performed once. Normoglycemia was defined as fasting blood glucose <100mg/dl and 2 hour blood glucose <140mg/dl. During the OGTT, blood pressure readings were obtained three times from all subjects in a sitting position. A food frequency questionnaire (EPIC-FFQ) for the assessment of dietary nutritional intake was completed online by the majority of the study participants (n = 87) [17]. In a subgroup of women (n = 62, consisting of 37 cases and 25 controls) whole body fat and abdominal subcutaneous and visceral fat were measured with an axial T1w or mDixon magnetic resonance imaging (MRI) technique [18] on a 3.0 Tesla scanner (Philips Healthcare, Best, The Netherlands). Liver fat was assessed with fat fraction maps generated from mDixon sequences [19].

### Blood sample collection and analysis

#### Analysis of serum NEFA

After 30 minutes of coagulation time, serum monovettes were centrifuged at 2000 g at room temperature. Serum aliquots were then immediately frozen on dry ice and stored at -80°C until analysis. The total NEFA concentrations were determined by an enzymatic calorimetric method (NEFA Kit, Wako Chemicals, Neuss, Germany). For quantification of the NEFA species, 20 μl of each serum sample was mixed with 200 μl isopropanol (containing the internal standard) in a 96-deepwell plate. After centrifugation, the supernatant was used for NEFA analysis by LC-MS/MS as previously described [20]. The NEFA were identified according to chain length and the number of double bonds. The positions of double bonds were estimated by the relative frequency of the isomeric NEFA in the fasting human blood [21]. Forty-one out of 47 NEFA fulfilled the quality control criteria that the coefficients of variation (CV) were below 20% for all measurements. Serum NEFA concentrations were calculated as molar percentages of the total NEFA [21]. Enzyme activities were estimated by the following ratios of products to substrates: 18:1/18:0 for stearoyl CoA desaturase-1 (SCD-1), 18:0/16:0 for elongation of very long chain fatty acids protein 6 (EloVL6), and 20:4/20:3 for delta-5-desaturase (Δ-5 D).

#### Laboratory measurements and calculations

Serum insulin was measured with chemiluminescence technology (CLIA, DiaSorin LIAISON systems, Saluggia, Italy). Glucose concentrations from sodium fluoride plasma were determined using a glucose analyzer (with the glucose oxidase method, Glucose HK Gen.3, Roche Diagnostics, Mannheim, Germany). The following additional laboratory parameters were analyzed: HbA1c (VARIANT II TURBO HbA1c Kit—2.0, Bio-Rad Laboratories, Hercules, USA), hsCRP (wide-range CRP, Siemens AG, Erlangen, Germany), gamma-GT (enzymatic caloric test, Roche Diagnostics), triglycerides (enzymatic caloric test, Roche Diagnostics), cholesterol (enzymatic caloric test, Roche Diagnostics), HDL (enzymatic caloric test, Roche Diagnostics), and LDL was calculated with the Friedewald equation (all triglyceride levels were below 400 mg/dl).

The following protein mediators were measured in plasma samples: fetuin-A (ELISA, BioVendor, Heidelberg, Germany), leptin (ELISA "Dual Range", Merck Millipore, Darmstadt, Germany), adiponectin (RIA, Merck Millipore), and resistin (Quantikine ELISA, R&D Systems, Wiesbaden-Nordenstadt, Germany).

The Matsuda Index was calculated as described previously [22] and used to quantify insulin sensitivity. Additionally, the HOMA-IR was assessed as the product of fasting glucose and fasting insulin concentrations, and the adipocyte insulin resistance (Adipocyte-IR) index was calculated as the product of fasting total NEFA_LC-MS/MS_ and fasting insulin concentrations [23, 24]. The disposition index was calculated from the OGTT to measure the relationship between insulin sensitivity and first-phase insulin secretion [25]. The rise in serum insulin during the first 30 minutes of the OGTT was used as a measure of the first-phase insulin secretion. The correlation of this measure with the first-phase secretion in the IVGTT was significantly better than that of the insulinogenic index in a substudy of the PPS-Diab study [15].

### Statistical analyses

The statistical analyses were performed with SPSS version 22.0 (SPSS Inc., Chicago, IL, USA). All numeric values are reported as median (first and third quartile) because the majority of the parameters were not normally distributed. Mann-Whitney U tests or a Chi-Square test were used to assess group differences. Multiple comparisons of the NEFA measurements were accounted for by Bonferroni correction. Those significant NEFA measurements from the Mann-Whitney U tests were further analyzed together with clinical parameters as independent variables by binary logistic regression models for the dependent variable pGDM status. The correlations between metric variables were calculated with Spearman's rank correlation coefficients (ρ) and with partial Spearman's rank correlation coefficients (ρ) with adjustment for BMI or total body fat volume. A p-value <0.05 was considered statistically significant. After Bonferroni correction alpha = 9.62e-4.

## Results

### Baseline characteristics

We included 62 normoglycemic women after a pregnancy complicated by GDM and 49 controls after a normoglycemic pregnancy in this analysis. The time from delivery to study inclusion was 3–16 months. The baseline characteristics of the study subjects are shown in **Table 1**. The Matsuda Index of insulin sensitivity and the Disposition Index were lower among the women after GDM (p = 0.030 and p = 0.002, respectively), but the HOMA-IR and the Adipocyte-IR Index were not different between the groups. Although all of the study subjects were normoglycemic, the glucose concentrations were significantly higher at each time point of the OGTT in post-GDM group. Metabolic risk factors such as triglycerides, HDL, hsCRP, gamma-GT, BMI, body fat percentage by bioimpedance measurement and WC were similar in the two groups. In an MRI substudy (n = 62), we also found no significant differences in total body fat volume, abdominal visceral fat volume and liver fat content. The hepatokine fetuin-A was significantly elevated in women after GDM (p = 0.008), but the serum concentrations of the adipokines leptin, adiponectin and resistin were not different (**Table 2**). There were also no significant differences in macronutrient consumption, as estimated by a food frequency questionnaire (EPIC-FFQ; **S1 Table**).

**Table 1:** Clinical and biochemical characteristics of the study cohort.

	post-GDM	controls	p-value
**Clinical characteristics**			
Months post-delivery	8.95 (7.50–11.60)	8.80 (7.00–10.30)	0.213
Hormonal contraception n (%)*	7 (11.3)	12 (24.5)	0.067
Age (years)	35 (32–38)	35 (32–37)	0.903
BMI (kg/m^2^)	23.2 (21.4–26.6)	22.7 (21.3–25.9)	0.297
WC (cm) (n = 58/49) ^#^	78 (73–86)	75 (71–83)	0.247
Waist to hip ratio (n = 58/49) ^#^	0.80 (0.76–0.86)	0.80 (0.75–0.84)	0.277
Body fat_BIA_ (%) (n = 62/48) ^#^	29.75 (25.70–37.70)	30.60 (26.20–35.20)	0.861
Systolic blood pressure (mmHg)	119 (112–125)	115 (107–123)	0.063
Diastolic blood pressure (mmHg)	75 (70–79)	72 (64–81)	0.068
Distance (m/d) (n = 41/41) ^#^	6610 (5104–8407)	6838 (5114–8259)	0.597
**MRI substudy** (n = 37/25) ^#^
Total fat_MRI_ (l)	20.25 (16.46–29.40)	19.65 (16.88–24.26)	0.571
Visceral fat_MRI_ ^a^ (l)	1.68 (1.13–2.84)	1.34 (1.01–1.91)	0.134
Subcutaneous fat_MRI_ ^a^ (l)	5.73 (4.12–8.29)	5.61 (4.31–6.72)	0.605
Liver fat (%)	0.51 (0.18–1.27)	0.41 (0.09–0.66)	0.137
**Biochemical characteristics**			
Matsuda Index	5.35 (3.68–8.02)	6.67 (4.84–8.51)	**0.030**
HOMA-IR	1.44 (0.94–2.35)	1.22 (0.81–1.91)	0.148
Adipocyte-IR Index^b^	3353 (1760–5496)	2901 (1911–4116)	0.290
Disposition Index	235.67 (176.73–303.60)	303.21 (244.65–368.26)	**0.002**
Fasting plasma glucose (mg/dl)	91 (87–95)	90 (83–92)	**0.026**
30min. plasma glucose (mg/dl)	151 (129–170)	140 (121–154)	**0.016**
60min. plasma glucose (mg/dl)	137 (113–163)	117 (99–132)	**0.001**
90min. plasma glucose (mg/dl)	111 (97–136)	100 (89–113)	**0.005**
2 hour plasma glucose (mg/dl)	112 (96–120)	94 (82–110)	**<0.001**
Triglyceride (mg/dl)	65 (51–89)	63 (50–87)	0.955
HDL (mg/dl)	64 (51–72)	64 (57–74)	0.570
LDL (mg/dl)	104 (89–115)	108 (97–123)	0.057
hsCRP (mg/dl)	0.06 (0.02–0.32)	0.04 (0.01–0.11)	0.252
Gamma-GT (U/l)	14 (12–19)	14 (11–18)	0.858
HbA1c (%)	5.4 (5.3–5.6)	5.3 (5.1–5.5)	**0.033**
HbA1c (mmol/mol)	35.5 (34.4–37.7)	34.4 (32.2–36.6)	**0.033**

The values are represented as the medians with interquartile ranges. If not stated otherwise n = 62 for the cases and n = 49 for the controls.

^*^Chi-Square test.

^#^(n = cases/controls).

^a^abdominal.

^b^Adipocyte-IR Index = fasting total NEFA_LC-MS/MS_ (μM) * fasting insulin (μU/ml).

**Table 2:** Plasma concentrations of selected biomarkers.

	post-GDM	controls	p-value
Fetuin-A (μg/ml)	288.35 (266.60–312.00)	263.40 (242.20–295.40)	**0.008**
Leptin (ng/ml)	8.73 (5.07–13.68)	6.89 (2.93–11.56)	0.071
Adiponectin (μg/ml)	9.99 (7.71–16.37)	11.63 (9.32–14.61)	0.512
Resistin (ng/ml)	8.52 (7.18–10.65)	8.90 (7.54–10.86)	0.845

The values are represented as the medians with interquartile ranges.

### Serum NEFA composition in women after GDM and controls

Total fasting NEFA concentrations did not differ between the women after GDM and controls (564.5 μM (407.0 μM—668.0 μM) vs. 548.0 μM (444.0 μM—681.0 μM), **Table 3**). The enzymatic measurement of serum NEFA and the calculated sum of all NEFA species as quantified by LC-MS/MS produced similar results (ρ = 0.86, p<0.001).

**Table 3:** Fasting serum NEFA profiles and total NEFA concentrations.

	post-GDM	controls	p-value*	BMI and HbA1c adj. p-value**
12:0	0.744% (0.595%-0.902%)	0.809% (0.720%-1.018%)	**0.008**	0.059
13:1	0.047% (0.034%-0.056%)	0.048% (0.039%-0.059%)	0.525	
14:0	3.013% (2.584%-3.457%)	3.346% (2.779%-3.775%)	**0.034**	**0.030**
14:1	0.617% (0.516%-0.720%)	0.607% (0.519%-0.755%)	0.913	
14:2	0.051% (0.043%-0.065%)	0.049% (0.043%-0.057%)	0.392	
15:0	0.495% (0.426%-0.596%)	0.542% (0.457%-0.598%)	0.238	
15:1	0.043% (0.036%-0.051%)	0.046% (0.038%-0.052%)	0.538	
16:0	25.112% (23.938%-25.857%)	25.615% (24.757%-26.314%)	**0.014**	**0.004**
16:1	5.338% (4.519%-6.021%)	4.982% (4.210%-5.781%)	0.230	
16:2	0.074% (0.066%-0.079%)	0.071% (0.062%-0.081%)	0.373	
16:3	0.026% (0.022%-0.031%)	0.026% (0.022%-0.029%)	0.723	
17:0	0.522% (0.473%-0.581%)	0.555% (0.507%-0.590%)	0.202	
17:1	0.357% (0.323%-0.387%)	0.342% (0.320%-0.394%)	0.553	
18:0	8.482% (7.664%-9.242%)	9.036% (8.410%-9.865%)	**0.034**	**0.033**
18:1	39.483% (38.272%-41.091%)	39.069% (37.352%-40.055%)	**0.040**	**0.019**
18:2	11.065% (10.236%-12.172%)	10.401% (9.865%-11.326%)	**0.034**	**0.019**
18:3	1.177% (1.031%-1.345%)	1.145% (1.071%-1.322%)	0.941	
18:4	0.025% (0.019%-0.032%)	0.028% (0.024%-0.036%)	0.077	
19:0	0.050% (0.044%-0.057%)	0.053% (0.046%-0.057%)	0.360	
19:1	0.136% (0.127%-0.150%)	0.141% (0.126%-0.155%)	0.658	
20:0	0.055% (0.048%-0.068%)	0.058% (0.051%-0.070%)	0.127	
20:1	0.349% (0.317%-0.398%)	0.344% (0.313%-0.387%)	0.517	
20:2	0.194% (0.169%-0.216%)	0.183% (0.166%-0.201%)	0.069	
20:3	0.215% (0.187%-0.234%)	0.208% (0.188%-0.232%)	0.931	
20:4	0.760% (0.616%-0.935%)	0.774% (0.617%-0.932%)	0.950	
20:5	0.089% (0.071%-0.114%)	0.090% (0.074%-0.108%)	0.739	
22:2	0.007% (0.006%-0.008%)	0.007% (0.006%-0.007%)	0.239	
22:3	0.010% (0.009%-0.011%)	0.010% (0.009%-0.011%)	0.890	
22:4	0.086% (0.077%-0.097%)	0.081% (0.071%-0.091%)	0.086	
22:5	0.180% (0.163%-0.202%)	0.183% (0.164%-0.204%)	0.766	
22:6	0.415% (0.359%-0.511%)	0.441% (0.345%-0.537%)	0.515	
24:0	0.024% (0.021%-0.030%)	0.027% (0.021%-0.032%)	0.262	
24:1	0.041% (0.034%-0.049%)	0.040% (0.034%-0.047%)	0.677	
24:3	0.002% (0.001%-0.002%)	0.002% (0.001%-0.002%)	0.442	
24:4	0.005% (0.005%-0.006%)	0.005% (0.004%-0.005%)	0.071	
24:5	0.007% (0.005%-0.008%)	0.006% (0.006%-0.008%)	0.928	
24:6	0.005% (0.004%-0.006%)	0.006% (0.005%-0.007%)	0.180	
26:0	0.002% (0.002%-0.004%)	0.003% (0.002%-0.004%)	**0.048**	0.979
26:1	0.006% (0.005%-0.007%)	0.006% (0.005%-0.007%)	0.901	
26:2	0.008% (0.007%-0.011%)	0.008% (0.006%-0.011%)	0.517	
26:3	0.003% (0.002%-0.004%)	0.003% (0.002%-0.004%)	0.633	
SFA	38.547% (36.940%-39.937%)	40.323% (38.769%-41.630%)	**<0.001** ^**#**^	**<0.001**
MUFA	46.655% (45.006%-48.549%)	45.972% (43.697%-47.010%)	**0.011**	**0.014**
PUFA	14.699% (13.652%-15.906%)	13.974% (13.295%-15.146%)	0.059	
∑ n-3	1.952% (1.733%-2.208%)	1.954% (1.801%-2.184%)	0.563	
∑ n-6	12.325% (11.493%-13.775%)	11.792% (11.004%-12.841%)	**0.033**	**0.015**
n-6/n-3	6.435% (5.819%-7.130%)	6.117% (5.283%-6.806%)	**0.020**	**0.045**
SCD-1 (18:1/18:0)	4.697 (4.164–5.317)	4.205 (3.908–4.747)	**0.010**	**0.013**
EloVL6 (18:0/16:0)	0.341 (0.315–0.373)	0.346 (0.318–0.384)	0.329	
Δ-5 D (20:4/20:3)	3.720 (3.058–4.314)	3.554 (3.253–4.119)	0.917	
Total NEFA_LC-MS/MS_	485.1 μM (402.8 μM -622.4 μM)	485.42 μM (408.1 μM -594.0 μM)	0.981	
Total NEFA_Enzymatic Test_	564.5 μM (407.0 μM -668.0 μM)	548.0 μM (444.0 μM -681.0 μM)	0.805	

Percentage concentrations (mol%) and absolute concentrations of NEFA are given as medians with interquartile ranges. n = 62 for the cases and n = 49 for the controls.

*Mann-Whitney U tests.

**Logistic regression analyses were adjusted for BMI and HbA1c when p<0.05 from Mann-Whitney U test.

#Significant after Bonferroni correction.

We were able to quantify 41 NEFA species by LC-MS/MS (**Table 3**). Monounsaturated fatty acids (MUFA) (46.4% (44.2%–47.8%) were predominantly present in fasting serum followed by SFA (39.5% (37.9%–40.8%) and PUFA (13.8% (13.0%–15.0%). The most abundant NEFA species were oleic acid (18:1 n-9), palmitic acid (16:0), linoleic acid (18:2 n-6) and stearic acid (18:0).

**Fig 1:**
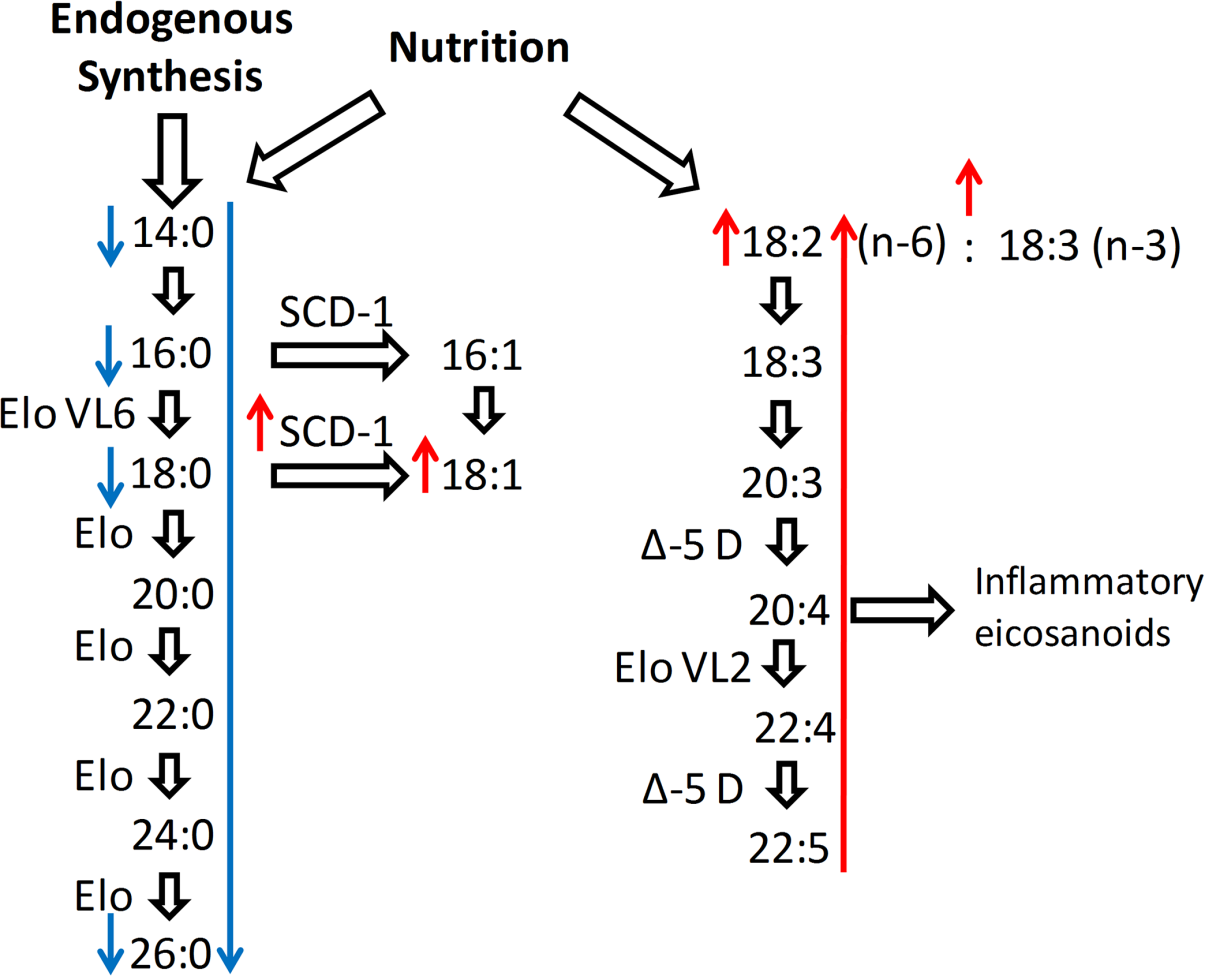
Altered NEFA pathways in cases compared to controls. At an exploratory level of significance (p<0.05), the women after GDM exhibited reduced levels of 12:0, 14:0, 16:0, 18:0, 26:0 and total SFA and elevated levels of 18:1, the essential fatty acid 18:2, total n-6 NEFA and the proportion of total n-6/n-3 NEFA. Calculated SCD-1 activity was significantly increased in the post-GDM group. Only total SFA remained significantly different after Bonferroni correction. The red arrows in the diagram represent upregulation, and the blue arrows represent downregulation in the post-GDM group.

At an exploratory level of significance (p<0.05) we found the following differences in the molar percentages of the various NEFA species in the post-GDM group compared to the controls: 12:0, 14:0, 16:0, 18:0, 26:0, and total SFA were reduced, and 18:1, 18:2, MUFA, total n-6 NEFA and the proportion of total n-6/n-3 NEFA were increased (**Table 3, Fig 1**). Cases also showed a higher calculated SCD-1 activity (4.70 vs. 4.21, p = 0.010, **Table 3**), but no changes in EloVL6 or Δ-5 D activities. All differences remained significant with adjustment for BMI and HbA1c, except for 12:0 and 26:0.

After Bonferroni correction for multiple testing, only total SFA were still significantly different between women after GDM and controls (38.6% vs. 40.3%, p = 0.0002). Total serum SFA were unrelated to the daily dietary intake of these fatty acids estimated from the EPIC-FFQ (**S2 Table**). We also did not observe a significant correlation with BMI, but found a negative association between SFA and estimated SCD-1 activity (ρ = -0.80, p<0.001).

### Associations of NEFA species with body composition and insulin resistance

We examined the associations between the different NEFA species and clinical parameters of body composition (BMI, WC) as well as total body fat and abdominal visceral adipose tissue from the MRI substudy. The group of women after GDM and the control group were combined for this analysis. All NEFA species with at least one association with a Spearman ρ≥0.3 are shown in **Table 4**. 16:3, 18:2, 22:4, 26:2 and 26:3 were positively associated with BMI and all other parameters of body fat, whereas 12:0, 14:0, 15:0, 20:0, 15:1, and 20:1 showed negative correlations. With respect to visceral adipose tissue, 14:0 and partially 12:0 remained significantly and inversely associated with WC and abdominal visceral adiposity after adjustment for BMI and total body fat, respectively (**Table 4**). The associations of NEFA species with measures of adiposity were similar in women after GDM and controls, when we repeated the analyses separately in the two groups (data not shown). We observed no relevant correlations with a Spearman ρ≥0.3 of single NEFA species and insulin sensitivity (Matsuda Index).

**Table 4:** Spearman correlation coefficients of selected fasting serum NEFA species with parameters of body composition.

	BMI	Body fat_BIA_	WC	WC (adjusted for BMI)	Total fat volume (MRI)	Abdominal fat volume (MRI)	Abdominal fat volume (adjusted for total fat volume, MRI)
**12:0**	-0.323***	-0.355***	-0.424***	-0.305**	-.225	-0.272*	-0.156
**14:0**	-0.372***	-0.417***	-0.480***	-0.340***	-0.473***	-0.533***	-0.290*
**15:0**	-0.206*	-0.279**	-0.273**	-0.192	-0.340**	-0.296*	-0.041
**15:1**	-0.202*	-0.244*	-0.278**	-0.209*	-0.256*	-0.314*	-0.188
**16:3**	0.287**	0.326***	0.290**	0.089	0.340**	0.285*	0.021
**18:2**	0.236*	0.210*	0.244*	0.083	0.285*	0.345**	0.202
**20:0**	-0.392***	-0.414***	-0.353***	-0.032	-0.451***	-0.363**	-0.001
**20:1**	-0.238*	-0.216*	-0.233*	-0.055	-0.340**	-0.308*	-0.062
**22:4**	0.312**	0.336***	0.343***	0.154	0.392**	0.412***	0.176
**26:2**	0.273**	0.326***	0.323***	0.178	0.342**	0.315*	0.072
**26:3**	0.297**	0.360***	0.386***	0.269**	0.261*	0.263*	0.093

All nonesterified fatty acid (NEFA) species with a Spearman correlation coefficient ρ≥0.3 with any of the listed parameters of body composition are shown in the table. The post-GDM and the control group were combined for this analysis. n = 106 for BMI, WC and percent body fat measured by BIA. n = 62 for the MRI substudy.

*Correlation is significant with p<0.05.

**Correlation is significant with p<0.01.

***Correlation is significant with p<0.001.

### Associations of essential fatty acids with dietary intake

Linoleic acid (18:2 n-6) and linolenic acid (18:3 n-3) are the only two fatty acids that cannot be synthesized endogenously. Over the whole study cohort, dietary intakes of 18:2 and 18:3 correlated with plasma levels of these fatty acids (ρ = 0.33, p = 0.002 and ρ = 0.23, p = 0.029, respectively). Furthermore, the sum of serum PUFA correlated with estimated PUFA intake (g/day) (ρ = 0.36, p = 0.001, **S2 Table**).

## Discussion

The main findings of this study are that in the fasting state the total serum NEFA concentration was similar in normoglycemic women with recent GDM and controls, but that differences between the groups existed in the NEFA composition. Women after GDM had a lower proportion of SFA and, at an exploratory level of significance, higher proportions of linoleic acid (18:2 n-6) and total n-6 NEFA. These differences were unchanged after adjustment for BMI and HbA1c. In a combined analysis of all study participants we also found positive as well as negative associations of several NEFA species with overweight/obesity. 14:0 and partially also 12:0 were inversely associated with abdominal visceral adiposity after adjustment for total body fat.

Women after GDM have an at least 10-fold increased risk of developing T2D later in life but are normoglycemic early after the index pregnancy in most cases [14]; thus, this population provides a unique opportunity to study an at-risk phenotype for T2D under normoglycemic conditions. This predisposition for T2D was also visible in our cross sectional data. Although the distributions of BMI, fat mass, liver fat, blood pressure, triglycerides and HDL/LDL cholesterol of the post-GDM group were similar to those of controls, the Matsuda Index and the Disposition Index were both significantly decreased. Hence, reduced insulin sensitivity and impaired beta-cell function were already present in these normoglycemic at-risk subjects.

Fasting serum NEFA are not directly derived from nutritional intake but primarily originate from adipose tissue storages [26, 27]. Thus, the association between the fatty acid composition of the adipose tissue and the NEFA profile in the fasting serum is strong [28]. Adipose tissue fatty acids are long-term markers of dietary fatty acid intake [21, 29] but also result from enzymatic modifications. Primarily, elongases and desaturases as well as enzymes involved in oxidation pathways affect fatty acid composition. The fatty acid-modifying enzymes for which connections to insulin resistance and T2D have been shown include delta-5, delta-6 and delta-9 desaturases (SCD-1) and elongase EloVL6 [30, 31]. SCD-1 is of particular interest because it is the rate-limiting step in the transformation of proinflammatory SFA to MUFA [32, 33].

The significant difference in the SFA proportion between women after GDM and controls that we observed was small (median 38.6% vs. 40.3%) but independent of BMI and HbA1c. Its biological relevance nevertheless remains uncertain. Our results suggest that the lower proportion of SFA in the post-GDM group was caused by endogenous transformation of SFA to MUFA and not by different nutritional intake. However, it is possible that the food frequency questionnaire did not adequately represent all aspects of fatty acid intake. Nevertheless the calculated activity of SCD-1 was elevated in the women after GDM and inversely correlated with the SFA proportion. Our approach to estimate SCD-1 activity has been shown to reflect subcutaneous adipose tissue SCD-1 expression [34]. We previously showed that elevated plasma fetuin-A, a predictive biomarker for T2D [35, 36], is also an independent risk marker in women with a recent history of GDM [15]. This finding was replicated in the present study. Since fetuin-A co-signals with SFA to induce adipose tissue inflammation [37], one possible explanation for the lower SFA proportions in normoglycemic women after GDM is that this constitutes a compensatory response to higher fetuin-A concentrations. However, at this point this remains speculative.

At an exploratory level of significance we also found higher levels of the essential fatty acid linoleic acid (18:2 n-6) and total n-6 NEFA in the post-GDM group. These NEFA species are linked to inflammatory signaling, insulin resistance and T2D risk [38, 39]. Nutritional advice for a 18:2-poor diet might therefore have the beneficial effect of less arachidonic acid (20:4 n-6) production and anti-inflammatory eicosanoid synthesis [40] in individuals at risk for T2D, in particular in women with GDM.

Correlations between distinct saturated fatty acids including 12:0, 14:0 and 18:0 and insulin sensitivity were previously shown in 70 year old men with established T2D by Iggman et al. [41]. We were unable to reproduce these observations, probably due to the different study populations. We found that the saturated fatty acids 12:0, 14:0, 15:0, 20:0, as well as the monounsaturated fatty acids 15:1 and 20:1, were inversely correlated with BMI and other parameters of adiposity in our study. 16:3, 18:2, 22:4, 26:2 and 26:3 showed positive correlations. These BMI-associated changes in the fasting serum NEFA might be related to pathologic alterations of the adipose tissue in obesity, e.g. adipocyte hypertrophy [42]. The SFA 14:0 and to a lesser extent 12:0 were particularly interesting with respect to body composition because they showed an inverse association with visceral adiposity in a BMI-independent manor. A low serum concentration of 14:0 has recently also been identified as a biomarker of T2D [43]. This link may be explained by the inverse association of 14:0 with visceral obesity that we saw in our analysis.

The major strength of our study is the consecutively recruited, normoglycemic cohort of young women with very few confounding diseases or medications. Limitations include the cross-sectional study design, which precludes propositions about causality and the prognostic relevance of our findings. Cohort size also limited the statistical power of some of our analyses. We also cannot exclude the possibility that our findings in part represent changes that are induced by the prior GDM or its treatment. However, nutritional information collected by food frequency questionnaire showed no differences between the post-GDM and the control group. Finally, it is unclear to what extent findings from a cohort of young women can be transferred to the general population.

In conclusion, this study shows differences in the fasting serum NEFA profile between women with a recent history of GDM, i.e. a high risk group for T2D, and control subjects. The NEFA composition also varied with overweight/obesity but not with insulin sensitivity. These findings are probably only in part related to fatty acid intake and mostly the result of enzymatic modifications. Our data should be confirmed in larger clinical cohorts and experimental evidence will be required to test their pathophysiological relevance.

## Supporting Information

S1 Table Group comparisons of the EPIC-FFQ data. (DOCX)

S2 Table Overview of the associations between fasting serum NEFA profiles and EPIC-FFQ data regarding the sums of the SFA, MUFA and PUFA. (DOCX)
