# Supplementary material for: Differences in the Serum Nonesterified Fatty Acid Profile of Young Women Associated with a Recent History of Gestational Diabetes and Overweight/Obesity
Source: PLoS One. 2015 May 26;10(5):e0128001. doi: 10.1371/journal.pone.0128001 (PMC4444334; doi:10.1371/journal.pone.0128001)
Supplement: S1 Table — (DOCX) [file pone.0128001.s001.docx]

**S1 Table:** Group comparisons of the EPIC-FFQ data.

| **n=47/40** | **post-GDM** | **controls** | **p-value** |
| --- | --- | --- | --- |
| Total foods (g/day) | 3598.77 (2997.40-3928.08) | 3494.80 (2775.47-3987.42) | 0.23 |
| Total energy (kJ/day) | 8455.99 (7557.40-9780.70) | 8439.30 (6598.60-10597.59) | 0.34 |
| Proteins (g/day) | 69.90 (61.42-78.04) | 66.07 (49.38-83-73) | 0.17 |
| Fat (g/day) | 86.71 (80.02-100.24) | 83.43 (67.42-114.50) | 0.385 |
| Carbohydrates (g/day) | 228.03 (187.49-252.72) | 212.52 (176.36-277.84) | 0.639 |
| Alcohol (g/day) | 3.36 (1.58-8.269 | 2.83 (1.55-9.86) | 0.63 |
| Dietary fiber (g/day) | 22.53 (18.06-27.14) | 21.55 (16.83-27.51) | 0.766 |
| Monosaccharides (g/day) | 40.39 (33.51-54.48) | 36.46 (28.15-47.90) | 0.093 |
| Fructose (g/day) | 21.67 (18.03-30.67) | 20.65(16.14-26.16) | 0.093 |
| Glucose (g/day) | 17.43(13.54-23.37) | 15.04 (11.95-19.89) | 0.096 |
| Disaccharides (g/day) | 66.85 (56.52-83.16) | 70.33 (55.90-83.78) | 0.733 |
| Polysaccharides (g/day) | 96.56 (78.30-115.70) | 90.38 (77.50-152.20) | 0.759 |
| 12:0 (g/day) | 2.02 (1.78-2.22) | 1.91 (1.55-2.68) | 0.821 |
| 14:0 (g/day) | 4.36 (3.68-5.42) | 4.20 (3.27-6.73) | 0.828 |
| 14:1 (g/day) | 0.51 (0.41-0.62) | 0.48 (0.37-0.79) | 0.905 |
| 15:0 (g day) | 0.42 (0.35-0.52) | 0.40 (0.31-0.67) | 0.925 |
| 15:1 (g/day) | 0.23 (0.19-0.29) | 0.23 (0.17-0.37) | 0.973 |
| 16:0 (g/day) | 17.48 (15.75-20.10) | 16.74 (13.07-24.59) | 0.506 |
| 16:1 (g/day) | 1.80 (1.62-2.26) | 1.82 (1.34-2.48) | 0.443 |
| 16:2 (g/day) | 0.001 (0.000-0.002) | 0.001 (0.000-0.002) | 0.556 |
| 17:0 (g/day) | 0.35 (0.29-0.43) | 0.33 (0.26-0.56) | 0.825 |
| 17:1 (g/day) | 0.35 (0.30-0.45) | 0.35 (0.27-0.57) | 0.898 |
| 18:0 (g/day) | 7.26 (6.58-8.57) | 7.06 (5.29-9.63) | 0.586 |
| 18:1 (g/day) | 28.17 (24.20-31.75) | 26.31 (20.13-35.44) | 0.285 |
| 18:2 (g/day) | 11.70 (9.97-13.89) | 10.66 (9.43-12.63) | 0.238 |
| 18:3 (g/day) | 1.74 (1.56-2.22) | 1.83 (1.34-2.24) | 0.892 |
| 18:4 (g/day) | 0.015 (0.004-0.021) | 0.017 (0.040-0.021) | 0.568 |
| 19:0 (g/day) | 0.004 (0.002-0.005) | 0.004 (0.003-0.007) | 0.218 |
| 20:0 (g/day) | 0.35 (0.32-0.41) | 0.33 (0.28-0.47) | 0.529 |
| 20:1 (g/day) | 0.46 (0.35-0.58) | 0.46 (0.34-0.55) | 0.805 |
| 20:2 (g/day) | 0.02 (0.01-0.03) | 0.02 (0.01-0.03) | 0.24 |
| 20:3 (g/day) | 0.04 (0.03-0.05) | 0.04 (0.03-0.06) | 0.667 |
| 20:4 (g/day) | 0.16 (0.13-0.19) | 0.15 (0.12-0.20) | 0.501 |
| 22:4 (g/day) | 0.001 (0.000-0.003) | 0.002 (0.000-0.003) | 0.55 |
| 22:5 (g/day) | 0.04 (0.03-0.05) | 0.04 (0.03-0.07) | 0.512 |
| 22:6 (g/day) | 0.18 (0.11-0.25) | 0.02 (0.02-0.03) | 0.885 |
| 24:1 (g/day) | 0.01 (0.00-0.01) | 0.01 (0.00-0.01) | 0.194 |
| SFA (g/day) | 36.30 (32.23-41.62) | 34.34 (27.05-52.04) | 0.597 |
| MUFA (g/day) | 31.69 (27.62-35.66) | 29.74 (22.81-40.48) | 0.293 |
| PUFA (g/day) | 13.89 (12.31-16.09) | 12.98 (11.33-15.49) | 0.307 |

The values are represented as the medians and interquartile ranges. n=47 for the post-GDM cohort and n=40 for the controls.
