# Supplementary material for: Differences in the Serum Nonesterified Fatty Acid Profile of Young Women Associated with a Recent History of Gestational Diabetes and Overweight/Obesity
Source: PLoS One. 2015 May 26;10(5):e0128001. doi: 10.1371/journal.pone.0128001 (PMC4444334; doi:10.1371/journal.pone.0128001)
Supplement: S2 Table — (DOCX) [file pone.0128001.s002.docx]

**S2 Table:** Overview of the associations between fasting serum NEFA profiles and EPIC-FFQ data regarding the sums of SFA, MUFA and PUFA in g/day. ^*^p=0.001

|  | **SFA (g/day)** | **MUFA (g/day)** | **PUFA (g/day)** |
| --- | --- | --- | --- |
| **SFA (mol%)** | 0.101 | -0.010 | -0.109 |
| **MUFA (mol%)** | 0.000 | -0.042 | -0.136 |
| **PUFA (mol%)** | -0.113 | 0.102 | 0.364^*^ |
